# Supplementary figures and images for: An improved similarity-based approach to predicting and mapping soil organic carbon and soil total nitrogen in a coastal region of northeastern China
Source: PeerJ. 2020 May 26;8:e9126. doi: 10.7717/peerj.9126 (PMC7258937; doi:10.7717/peerj.9126)

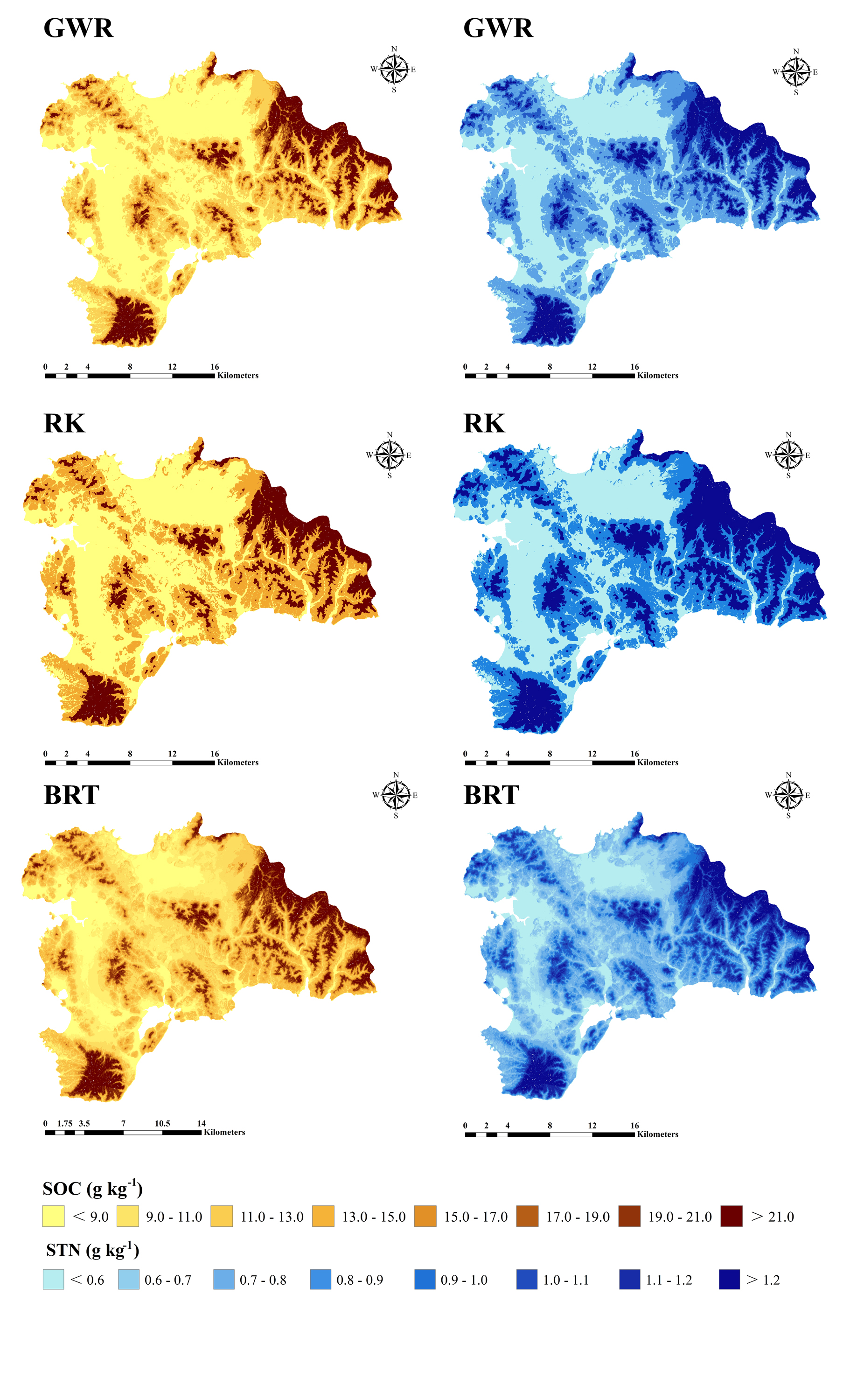

Supplement: Figure S1 [file peerj-08-9126-s002.jpg]
